# Hypertrophic Osteoarthropathy Mimicking Rheumatoid Arthritis

**DOI:** 10.7759/cureus.71153

**Published:** 2024-10-09

**Authors:** Alina Abid, Umer Nawaz, Kehinde O Sunmboye

**Affiliations:** 1 Internal Medicine, University Hospitals of Leicester National Health Service Trust, Leicester, GBR; 2 Internal Medicine, National Health Service England Education East Midlands, Leicester, GBR; 3 College of Health Sciences, University of Leicester, Leicester, GBR; 4 Rheumatology, University Hospitals of Leicester National Health Service Trust, Leicester, GBR

**Keywords:** hypertrophic osteoarthopathy, inflammatory arthritis, lung cancer, paraneoplastic rheumatologic syndromes, rheumatoid-like

## Abstract

Paraneoplastic rheumatologic syndromes encompass a range of clinical conditions that mimic primary rheumatic diseases and occur in the context of malignancy. Hypertrophic osteoarthropathy (HOA) is a notable example of such a syndrome, which is frequently associated with intrathoracic malignancies, including primary lung tumors and metastases.

This report presents a case of a patient diagnosed with HOA secondary to lung adenocarcinoma, who was admitted with symmetric polyarthritis that resembled elderly-onset rheumatoid arthritis. Anti-cyclic citrullinated peptide (anti-CCP) antibodies are primarily associated with rheumatoid arthritis, but their levels can be falsely elevated in various other conditions. In clinical practice, it’s essential to interpret anti-CCP antibody results in conjunction with other clinical findings and laboratory tests to arrive at an accurate diagnosis.

This case underscores the importance of considering HOA in the differential diagnosis of inflammatory arthritis, particularly in patients with a known or suspected malignancy, especially in the presence of positive rheumatoid arthritis-specific antibodies. Also, recognition and treatment of the underlying condition can lead to substantial improvements in both rheumatologic and oncologic aspects.

## Introduction

This case report focuses on the complexities of diagnosing inflammatory polyarthritis in elderly patients, where several clinical conditions must be considered. Among these are polyarticular gout, pseudogout, elderly onset rheumatoid arthritis (RA), osteoarthritis, and paraneoplastic syndromes. Paraneoplastic rheumatologic syndromes are particularly noteworthy, as they present clinical features that resemble primary rheumatic diseases, yet are associated with underlying malignancies. These syndromes often show improvement with effective treatment of the cancer [1].

One significant paraneoplastic syndrome is hypertrophic osteoarthropathy (HOA), which is characterized by a triad of clinical manifestations: periostosis, digital clubbing, and soft tissue swelling in the distal extremities [2]. HOA is most frequently linked to intrathoracic malignancies, particularly non-small cell lung cancer, which tends to have a poor prognosis. Understanding the interplay between these conditions is crucial for timely diagnosis and management in elderly patients presenting with inflammatory polyarthritis [3].

## Case presentation

A 74-year-old gentleman with a significant 50-pack-year smoking history and a past medical history that included paroxysmal atrial fibrillation, peripheral vascular disease, and type 2 diabetes mellitus presented for evaluation. He reported a 10-month history of persistent pain localized to the left big toe joint. This joint pain was accompanied by notable weight loss and increasing shortness of breath over the past few months.

Upon examination, the patient exhibited digital clubbing, which raised suspicion for an underlying pulmonary condition [4]. Notably, there was no evidence of synovitis or restriction in joint movements, which helped differentiate his symptoms from other inflammatory conditions.

**Figure 1:**
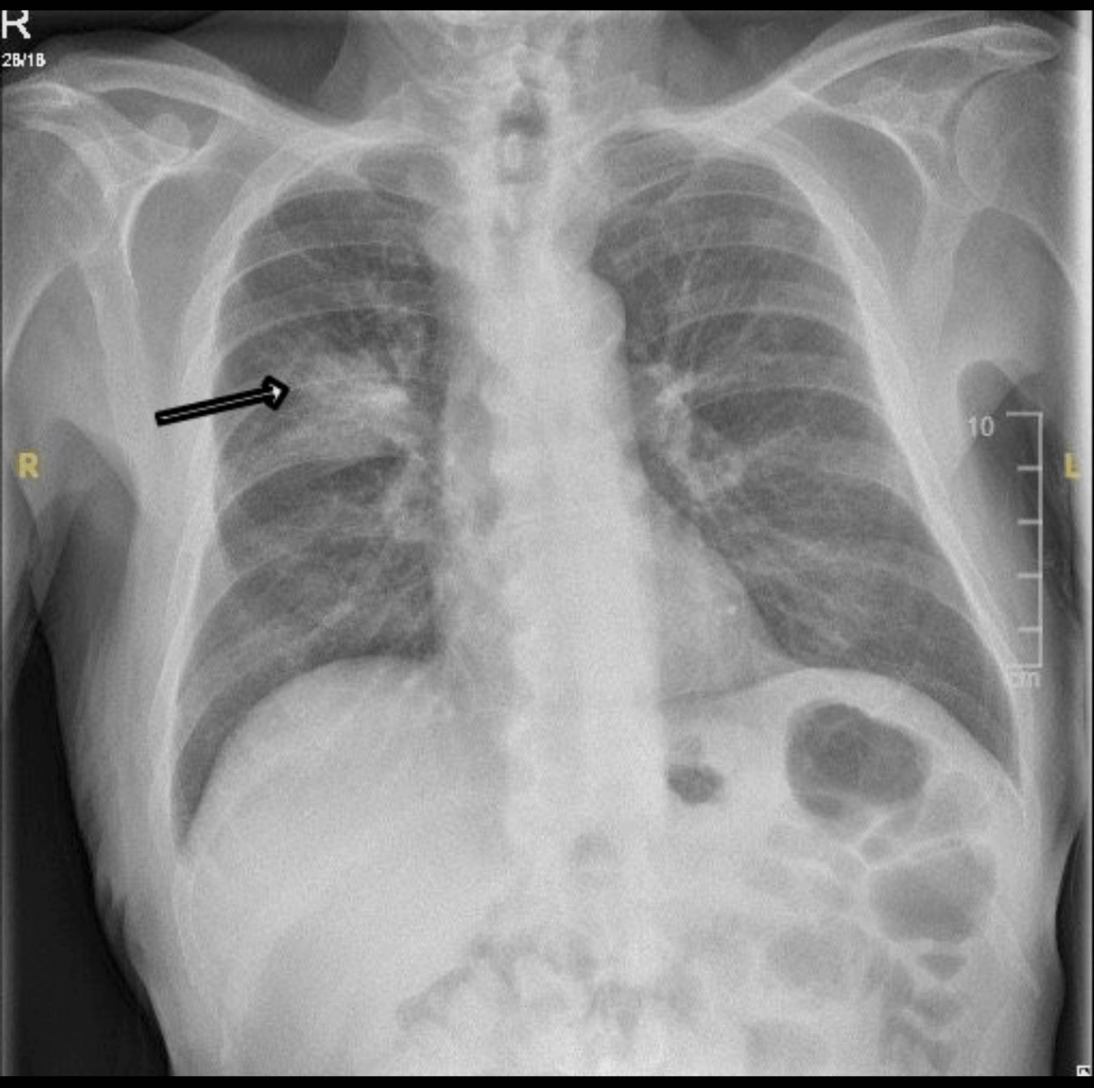
Posteroanterior chest radiography The image shows a right perihilar mass (arrow)

Laboratory investigations revealed significantly elevated rheumatoid factor at 1300 (normal range: 0-15) and anti-cyclic citrullinated peptide (anti-CCP) antibodies greater than 340 (normal range: 0-6), indicating a strong autoimmune component. Additionally, his C-reactive protein (CRP) level was elevated at 54, suggesting an inflammatory process. A chest X-ray was performed, which indicated a right middle zone mass, prompting further investigation (Figure 1).

**Figure 2:**
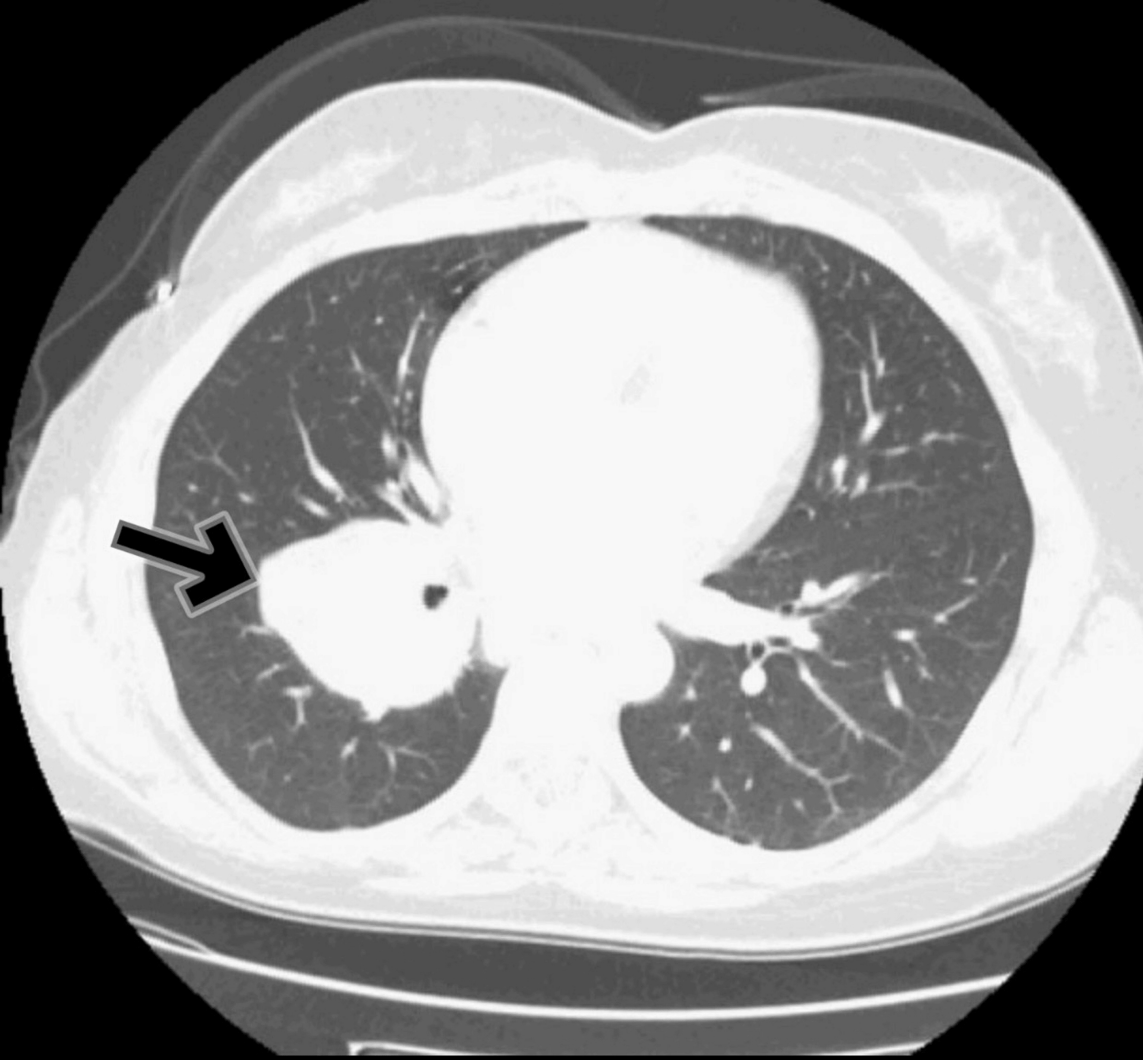
CT scan of the thorax Confirmed right perihilar mass (arrow)

A CT scan of the thorax, abdomen, and pelvis was conducted to assess the mass and evaluate for any potential malignancies, given the patient's age, smoking history, and clinical presentation, which concluded as a right perihilar mass with confluent mediastinal and right hilar lymphadenopathy, highly suspicious for primary lung carcinoma (Figure 2).

This case highlights the importance of thorough evaluation in elderly patients presenting with joint pain and associated systemic symptoms, particularly in the context of significant risk factors for malignancy. The combination of elevated rheumatoid markers, digital clubbing, and the presence of a pulmonary mass strongly suggests a paraneoplastic process, warranting urgent intervention and management.

## Discussion

HOA can present with clinical features that closely resemble those of RA, making differential diagnosis challenging [5]. In this case, the patient exhibited elevated rheumatoid factor (RF) and anti-CCP antibodies, both of which are commonly used in the diagnosis of RA. The pooled sensitivity of RF is approximately 69%, with a specificity of 85%, while anti-CCP antibodies demonstrate a sensitivity of 66% and a specificity of 90.4%. While these serological markers are valuable in diagnosing RA, they can yield false-positive results, particularly in certain populations, leading to potential misdiagnosis [6]. The differential diagnosis of polyarthritis in elderly patients includes RA, polyarticular gout, inflammatory osteoarthritis, polymyalgia rheumatica, and paraneoplastic syndromes such as HOA. Distinguishing between these conditions can be difficult, particularly when serological markers like RF and anti-CCP are positive, as in this case. Anti-CCP antibodies have a specificity of 90% for RA but can be falsely elevated in other conditions, including malignancies [7].

Notably, studies have indicated a correlation between smoking and elevated levels of RF and anti-CCP antibodies. In heavy smokers, the antibody titers are often significantly higher, suggesting that smoking may influence the autoimmune response. This relationship complicates the interpretation of serological results in patients with a significant smoking history, as seen in our patient [8,9].

The presence of digital clubbing and an elevated CRP in a heavy smoker should prompt consideration of HOA, especially if there is radiographic evidence of a pulmonary mass. The pathophysiology of HOA is not fully understood but is thought to involve the release of growth factors such as vascular endothelial growth factor (VEGF) from the tumor, leading to periosteal and soft tissue changes. This paraneoplastic process underscores the complex interaction between malignancy and systemic inflammation, which can result in clinical features that overlap with primary autoimmune diseases.

This case serves as a critical reminder that HOA can mimic RA, especially when the clinical presentation lacks definitive signs of inflammatory arthritis. The presence of red flags such as a history of heavy smoking and digital clubbing should heighten clinical suspicion for potential paraneoplastic syndromes [10]. In instances where the typical features of inflammatory arthritis are not convincingly present, a thoughtful evaluation is essential to rule out underlying malignancies [11].

A high index of suspicion is warranted, particularly in elderly patients with risk factors for cancer. Clinicians should consider comprehensive imaging and further investigations to explore potential underlying conditions, such as intrathoracic malignancies, when faced with atypical presentations of joint pain and systemic symptoms. Ultimately, accurate diagnosis and timely intervention are crucial for improving patient outcomes and guiding appropriate treatment strategies [12]. The mainstay of treatment for paraneoplastic HOA is addressing the underlying malignancy. Non-steroidal anti-inflammatory drugs (NSAIDs) can provide symptomatic relief, but long-term control of HOA hinges on successful cancer treatment. There are emerging therapies targeting VEGF and other growth factors implicated in the pathogenesis of HOA, though their clinical use remains investigational.

## Conclusions

HOA must be considered in the differential diagnosis of inflammatory arthritis, particularly in elderly patients presenting with joint pain and systemic symptoms. This case demonstrates the diagnostic complexity of HOA mimicking RA in an elderly patient with lung adenocarcinoma. It underscores the need for clinicians to maintain a high index of suspicion for paraneoplastic syndromes in patients with unexplained polyarthritis and cancer risk factors. It highlights that elevated RF and anti-CCP antibodies are typically associated with RA but can be misleading in patients with malignancies, especially those who smoke.
